# Effectiveness of mDiabetes intervention in enhancing diabetes awareness and promoting healthy lifestyle changes among the general population in rural India

**DOI:** 10.3389/fpubh.2024.1470615

**Published:** 2025-01-29

**Authors:** Padmaja Kumari Rani, Jachin David Williams, Nidhi Jaswal, Venkateswaralu Yandluri, Payal Sangani, Kavya Sanagavarapu, Ramya Natarajan, Sandhya Ramalingam, Nalini Saligram, Rohit C. Khanna

**Affiliations:** ^1^Department of Teleophthalmology, L V Prasad Eye Institute, Hyderabad, Telangana, India; ^2^Department of Vitreoretina, Anant Bajaj Retina Institute, L V Prasad Eye Institute, Hyderabad, Telangana, India; ^3^School of Medicine and Dentistry, University of Rochester, Rochester, NY, United States; ^4^Allen Foster Community Eye Health Research Centre, Gullapalli Pratibha Rao International Centre for Advancement of Rural Eye Care, L V Prasad Eye Institute, Hyderabad, India; ^5^Arogya World, Spring House, PA, United States; ^6^Suven Clinical Research Centre, L V Prasad Eye Institute, Hyderabad, India; ^7^Ophthalmic Biophysics Laboratory, L V Prasad Eye Institute, Hyderabad, India; ^8^Brien Holden Eye Research Centre, L.V. Prasad Eye Institute, Hyderabad, India; ^9^School of Optometry and Vision Science, University of New South Wales, Sydney, NSW, Australia

**Keywords:** voice messages, diabetes awareness, lifestyle changes, community health education, behavior changes

## Abstract

**Aim:**

This study aimed to assess the impact of a mHealth and community health education intervention on diabetes awareness and promoting healthy dietary and lifestyle habits within a rural population in Andhra Pradesh, India.

**Methods:**

Using a quasi-experimental design, the mDiabetes program was implemented for 1 year, among 1,03,538 rural individuals. Under this program, 56 diabetes prevention messages (twice a week) in local language) were disseminated among the participants via voice calls for a period of 6 months. Additionally, community health education meetings were facilitated by trained community health workers and educational leaflets were distributed among the community members. Questionnaires were administered at three different time points-baseline (before the intervention), endline (after intervention), and follow-up (3 months after endline) to collect demographic data, diabetes-related knowledge, attitudes, practices, physical activity, and dietary habits. Analysis compared data from 545 subjects who participated in all the three surveys.

**Results:**

The cohort comprised 45.5% males and 54.5% females, aged 19–85 years (mean: 55.42; SD 10.3). Post-intervention, diabetes awareness rose to 97.43% at endline and 99.63% at follow-up from 82.75% at baseline. Belief in diabetes preventability increased from 25.5% to 69.5%, and awareness of lifestyle's impact on diabetes management improved from 72.6% to 80.9%. Over 90% recalled prevention messages, with significant lifestyle changes reported by 83% at endline and 73% at follow-up. Improved dietary and activity habits were evident, with fruit consumption and high-fat food avoidance at 78.5% and 67.7% in follow-up. Physical activity levels improved in both endline and follow-up groups compared to baseline. Daily participation in yoga, running, gym, and aerobics increased to 38.7% in endline and follow-up from 7.3% at baseline (*p* < 0.001). Outdoor sports engagement rose significantly to 15% in endline and follow-up from 0.5% at baseline (*p* < 0.001). Regular stair usage (59.8%), walking for chores (84.7%), and short walking breaks (93%) increased significantly in follow-up compared to baseline and endline (*p* < 0.001).

**Conclusion:**

The combined mHealth and community health education intervention improved diabetes awareness and healthy habits in rural areas, showing potential for lasting outcomes and guiding future public health efforts in similar settings.

## Introduction

India has emerged as a major hub of diabetes cases, affecting both urban and rural regions.

The study conducted by the ICMR-INDIAB group has revealed that in India, more than 101 million individuals aged 18 and above are living with diabetes. In addition, there are about 135 million adults who are considered to be in a pre-diabetic state (1). A recent pooled systematic review and meta-analysis of 1.7 million adults showed prevalence of diabetes increased in both rural and urban India from 2.4% and 3.3% in 1972 to 15.0% and 19.0%, respectively, in the year 2015–2019 (2). These increasing trends of narrow urban and rural divide and equal gender affliction are alarming and can be attributed to changes in dietary and physical activity habits, along with economic growth patterns (2). The rising diabetes burden has led to significant morbidity, mortality, and economic implications due to associated micro and macrovascular complications (3, 4). Moreover, the indirect cost from premature death due to diabetes was over 64% in Low middle Income countries (LMICs) and 60% in high Income Countries [HICs; (5)]. Individuals with diabetes in LMICs tend to die at a younger and more productive age than do people with diabetes in HICs (5). Urgent measures are required to address this growing health crisis and its socio-economic consequences. Community-based programs using technology have proven to be highly effective in reaching a diverse audience, especially in areas with limited healthcare resources and restricted access to medical services (6).

Global experts in diabetes prevention recommend the establishment of diabetes prevention programs led by non-physician personnel, utilizing technology, and community education (7). Mobile health (mHealth) technology, particularly in the form of text messaging, has shown great promise in preventing diabetes and its complications, facilitating behavior change in low/middle income countries (7, 8). With a significant number of mobile users in India, mHealth technology (mobile interventions) for the prevention of diabetes and diabetes-related complications is a promising option. Diabetes prevention messages delivered via SMS, personalized text messages, mobile apps, television-based lifestyle interventions, and weekly coaching calls have demonstrated a significant positive impact on the primary prevention of diabetes and the monitoring of diabetes control (8–10). However, these studies predominantly focused on users from urban locations.

There is a need to study the effectiveness of such mHealth interventions in rural areas. Additionally, community health education, implemented through community health workers in LMICs, has demonstrated positive impacts on diabetes prevention and control (11).

Combining these approaches, the present study aims to assess the effectiveness of a combined mHealth (diabetes prevention messages over voice calls) and community health education intervention, delivered by community health workers, in raising awareness about diabetes, promoting dietary and lifestyle changes among a rural population cohort in the southern state of Andhra Pradesh, India.

## Methods

### Study design

mDiabetes program is a 1 year, community-based intervention with a pre-test and post-test, quasi-experimental design. The study was approved by the institutional review board (LEC-BHR-P-06-21-665). Each participant provided verbal informed consent, and the study followed the Declaration of Helsinki.

### Study population

The baseline assessment, conducted in October and November 2021, involved 1,019 subjects (The study recruitment period was between October 1st 2021–November 30th 2021). Among these participants, participants who expressed willingness to continue were included in the endline assessment and follow-up survey, conducted in November 2022 and February 2023, respectively. The inclusion criteria included participants who were 18 years or older, mobile users from the LV Prasad Eye Institute (LVPEI) patient database within the project area, and who were willing to participate by providing informed verbal consent. We initially approached 1,019 subjects from a larger database of 103,538 individuals, anticipating a minimum of 500 participants to complete all three surveys (baseline, endline, and follow-up). The endline survey (*n* = 710) was conducted immediately after the intervention at 6 months, while the follow-up survey (*n* = 625) was conducted 3 months after endline survey. Analysis compared data from 545 subjects who participated in all three surveys. Comparative analysis of baseline responses between participants (*n* = 545) and non-participants (*n* = 474) showed similarity, with no significant differences observed (Supplementary Table 1).

### Intervention design and implementation

Arogya World's mDiabetes program is an mHealth solution designed to provide diabetes prevention and control information through messages sent to the mobile phones of people-regardless of their risk status. In collaboration with the Rollins School of Public Health at Emory University, 56 mDiabetes messages were developed in 2011 based on the transtheoretical model of behavior change (8).

The content was made available in 12 languages and was reviewed by Arogya World's Behavior Change Task Force, which consisted of national and international experts in diabetes research, public health and behavior change. These 56 messages are delivered as automated voice calls (twice a week) for 6 months in the current study. These messages, categorized into five key themes, focused on medical information (20 messages), lifestyle changes (8 messages), nutrition (13 messages), fitness and physical activity (9 messages), and motivational content (6 messages). The messages provided practical advice on managing diabetes complications, adopting healthy habits such as balanced nutrition, quitting smoking, and engaging in daily physical activity like walking and yoga. Each message was concise, with a character range of 84–160 characters, ensuring clarity and accessibility. The content was designed in simple, actionable language to encourage participants to make sustainable lifestyle changes and improve diabetes awareness (examples of message content—Supplementary File 2).

During the registration process, participants were instructed to save the mobile number from which they would receive voice calls under the name “Arogya mDiabetes” on their phones to ensure they recognized the calls. If a mobile phone was unreachable or a participant did not respond, the scheduled voice call was reattempted at two different subsequent times.IMI Mobile was the technology partner for delivering automated mDiabetes voice calls. Pre-recorded content, tested for accuracy and reliability, was sent in Telugu between 4 and 7 p.m. over 6 months. If a call was missed, two retries were made at 5 min intervals.

The decision to send messages twice a week was based on balancing effective intervention delivery with participant engagement and adherence, particularly in rural settings. Research indicates that overly frequent messaging can cause fatigue, reducing intervention effectiveness while infrequent messaging may fail to sustain behavioral changes (12). This frequency also considers participant time constraints, mobile data usage, and communication preferences, making it an optimal choice for maintaining engagement without overwhelming recipients.

**Figure 1:**
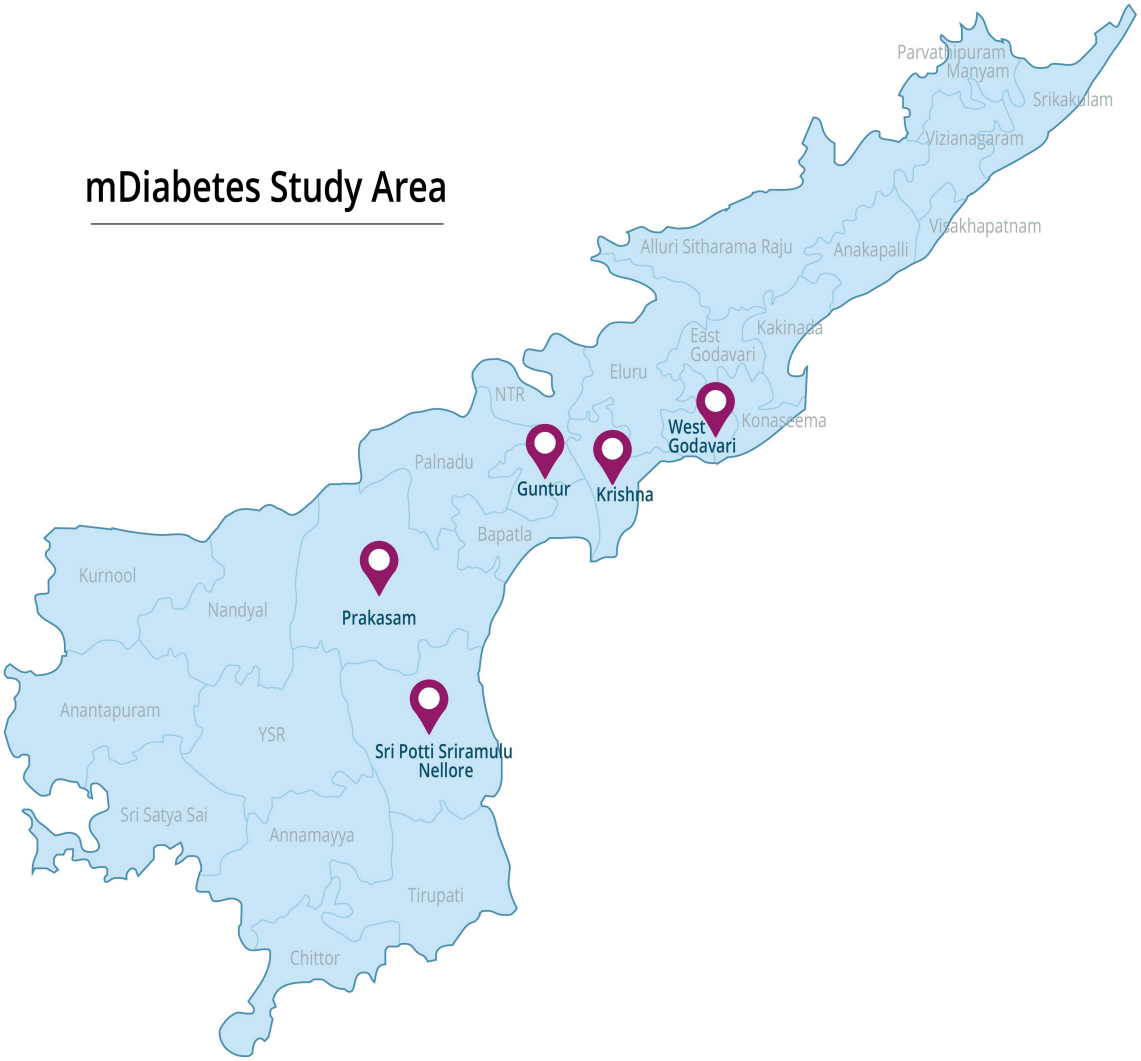
Project area map.

**Figure 2:**
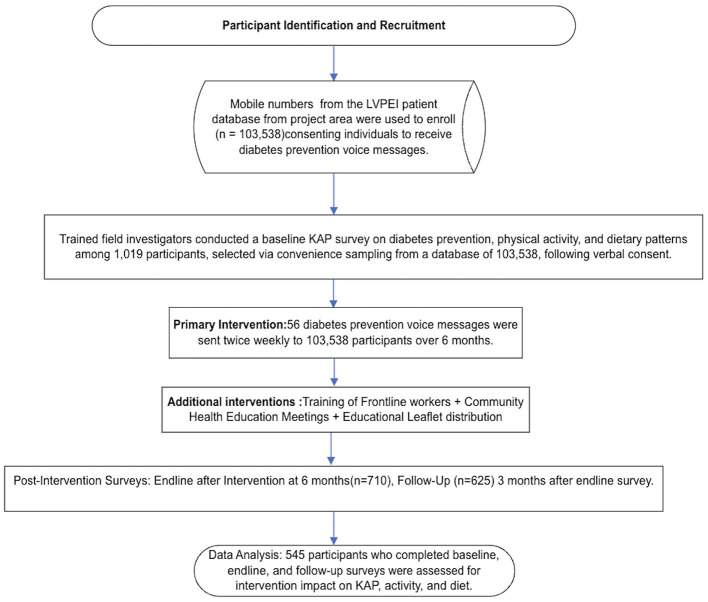
Study flow chart.

The mDiabetes project employed mHealth technology to disseminate 56 diabetes prevention messages in the regional language (Telugu) via voice calls to over 1,03,538 individuals over a 6 month period. Study participants were recruited from the pool of mDiabetes project subjects in Andhra Pradesh's districts of Krishna, Nellore, East Godavari, and Krishna (Figure 1 shows the project area map). The study methodology is comprehensively illustrated in Figure 2: Study Flow Chart.

To ensure effective dissemination, around 400 frontline workers (ASHA (Accredited Social Health Activists) were trained in delivering diabetes prevention messages. The training of frontline workers (ASHA) was conducted systematically on online platforms due to the COVID-19 pandemic, equipping them with the knowledge and skills to deliver diabetes prevention messages effectively. The content was structured into four modules: Module I focused on understanding diabetes, its types, risk factors, and symptoms; Module II addressed diabetes-related complications (retinopathy, neuropathy, nephropathy, and cardiovascular diseases) and their management; Module III emphasized healthy eating, promoting a balanced diet with whole grains, proteins, fruits, and vegetables while limiting sugar, salt, and fat; and Module IV highlighted the importance of physical activity, recommending 150 min of weekly exercise and strategies to overcome barriers. Training materials, including flip charts and educational leaflets, were translated into regional languages and validated for accuracy to ensure effective communication.

A total of 136 community health education meetings were conducted by trained frontline workers to reinforce key diabetes prevention messages, reaching 5,681 participants (2,393 males and 3,288 females). The sessions focused on practical strategies, including understanding diabetes, its risk factors and symptoms, preventing complications, adopting management strategies, promoting healthy eating habits, and encouraging regular physical activity while addressing barriers. Flip charts and translated educational materials were used to enhance engagement and understanding, and 10,000 educational leaflets on diabetes prevention (sample leaflets enclosed—Supplementary File 3) were distributed across the project area to further reinforce the messages.

Events were held to celebrate World Diabetes Day in the project area, further promoting awareness about diabetes and its potential complications. Diabetes prevention videos were thoughtfully played in LV Prasad Eye Institute centers targeting patients and their attendants as a part of the education campaign. The field investigators collected periodic feedback from the residents in the field area, ensuring valuable insights for continuous improvement.

### Data collection: KAP assessment

Field investigators have been provided with phone numbers and addresses of participants from the LVPEI patient database (General population) who received M DIABETES m-health intervention in the form of audio messages. Investigators employed a convenience sampling strategy, reaching out to participants at their study locations and recruiting those who expressed willingness to participate in the study. A convenience sampling strategy was chosen for logistic reasons and to accommodate the constraints of public health settings.

The questionnaires administered at three time points: baseline (before the intervention), endline (immediately after the intervention at 6 months)aft and follow up (3 months after endline survey) collected demographic information, knowledge, attitudes, and practices related to diabetes, physical activity, and dietary habits. The endline questionnaire included additional questions about the impact of diabetes prevention awareness messages on lifestyle changes. The follow-up questionnaire aimed to assess the effect of community educational interventions. Field investigators received comprehensive training on the proper administration of questionnaires to ensure consistency and reliability in data collection, which involved the use of Google Forms with timestamps. Furthermore, to verify the accuracy of the responses, a validation process was implemented, wherein entries from each investigator were systematically audited for correctness and completeness. The collected data provided insights into the effectiveness of diabetes prevention awareness messages and their impact on lifestyle modifications among the study participants.

### Statistical analysis

Statistical data analysis was conducted in 2023 through rigorous coding and cleaning of the collected datasets. One thousand and nineteen participants responded to the baseline survey, and 545 of them consistently responded in the two subsequent post-intervention surveys. Participant responses were analyzed using descriptive statistics (frequency and proportion) and inferential statistical methods in IBM SPSS Version 21 software. We applied a paired *t*-test to assess the statistical differences between the pre- and post-intervention endline and follow-up survey responses. We used independent-test for the age and χ^2^ test for dichotomous variables to observe the baseline characteristics of follow-up visit participants and non-responders group (Supplementary material 1). Additionally, we ran ANOVA test to observe the influence of demographic and socio-economic factors (i.e., gender, age, education, and occupation) on intervention outcomes. A significance level of *p*-value set at 0.05 to interpret the intervention outcomes.

## Results

### Study population characteristics

The final analysis involves a comprehensive comparison of data from the same 545 subjects who actively participated in the baseline, endline, and follow-up surveys. The study cohort consisted of 45.5% males and 54.5% females, with an age range spanning from 19 to 85 years, and a mean age of 55.42; SD 10.3 years. Regarding education, 7.9% (*n* = 43) completed higher education, 47.7% (*n* = 260) completed 10 years of schooling, and 44.4% (*n* = 242) either had no formal education or it was not applicable to them. Occupationally, 52.2% (*n* = 286) were employed in office jobs, 18.3% (*n* = 100) were skilled laborers, 22.2% (*n* = 121) were unskilled laborers, 4.6% (*n* = 25) were business owners, and 2.7% (*n* = 13) were unemployed.

### Awareness—DM

During the baseline assessment, a notable 82.75% of the participants demonstrated awareness of diabetes. However, following the intervention, there was a significant increase in awareness levels. In the endline assessment, 97.43% reported being aware of information related to diabetes prevention and this awareness further improved to 99.63% in the follow-up survey.

**Figure 3:**
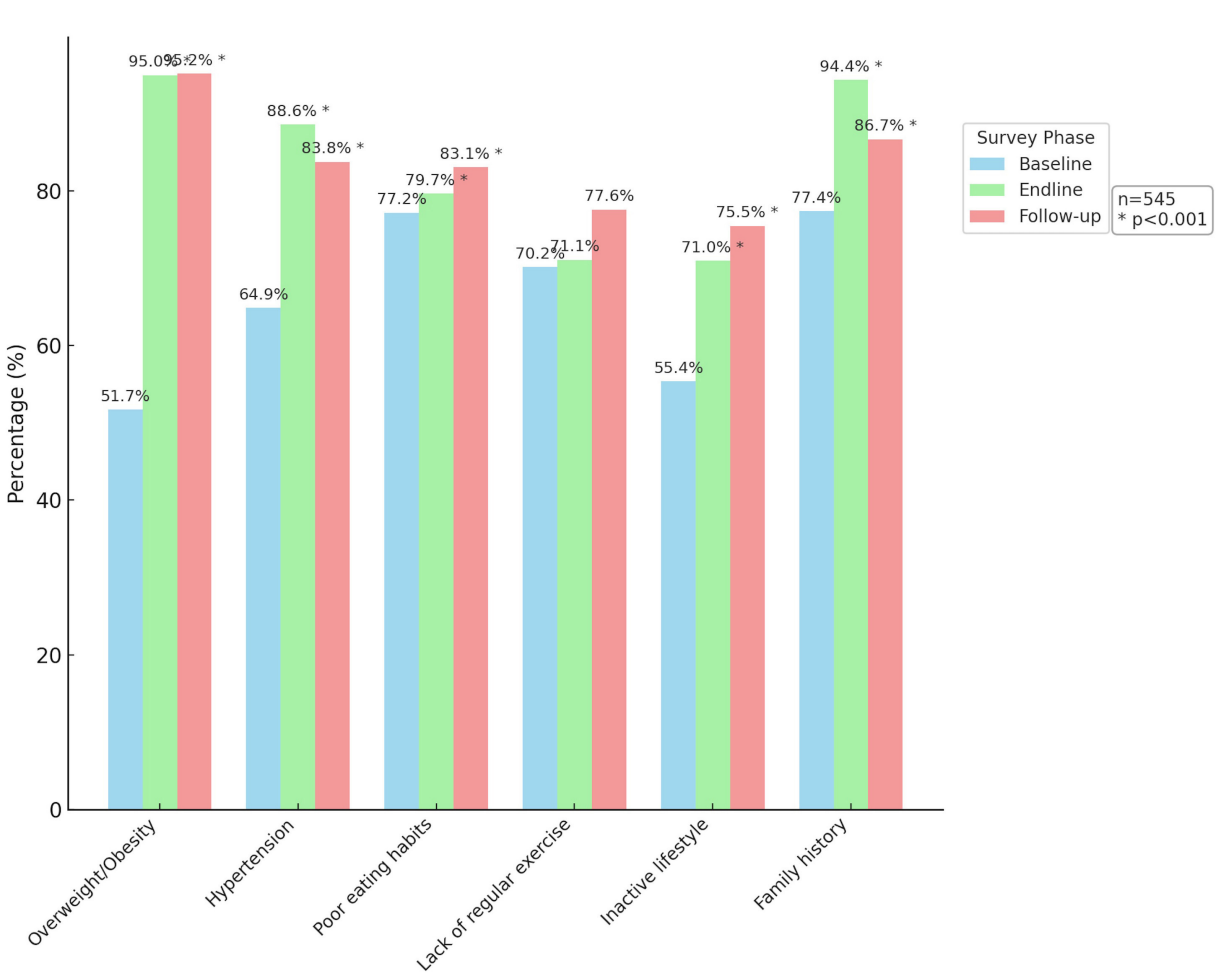
Awareness about the causes of diabetes.

Figure 3 illustrates the results of the analysis, focusing on respondents who indicated a positive response (i.e., “Yes”) for each question. The intervention had a significant impact on enhancing awareness about diabetes causes, particularly in relation to overweight/obesity, hypertension, poor eating habits, inactive lifestyle, and family history. However, it is noteworthy that awareness of the role of lack of regular exercise remained relatively unchanged despite the intervention.

**Figure 4:**
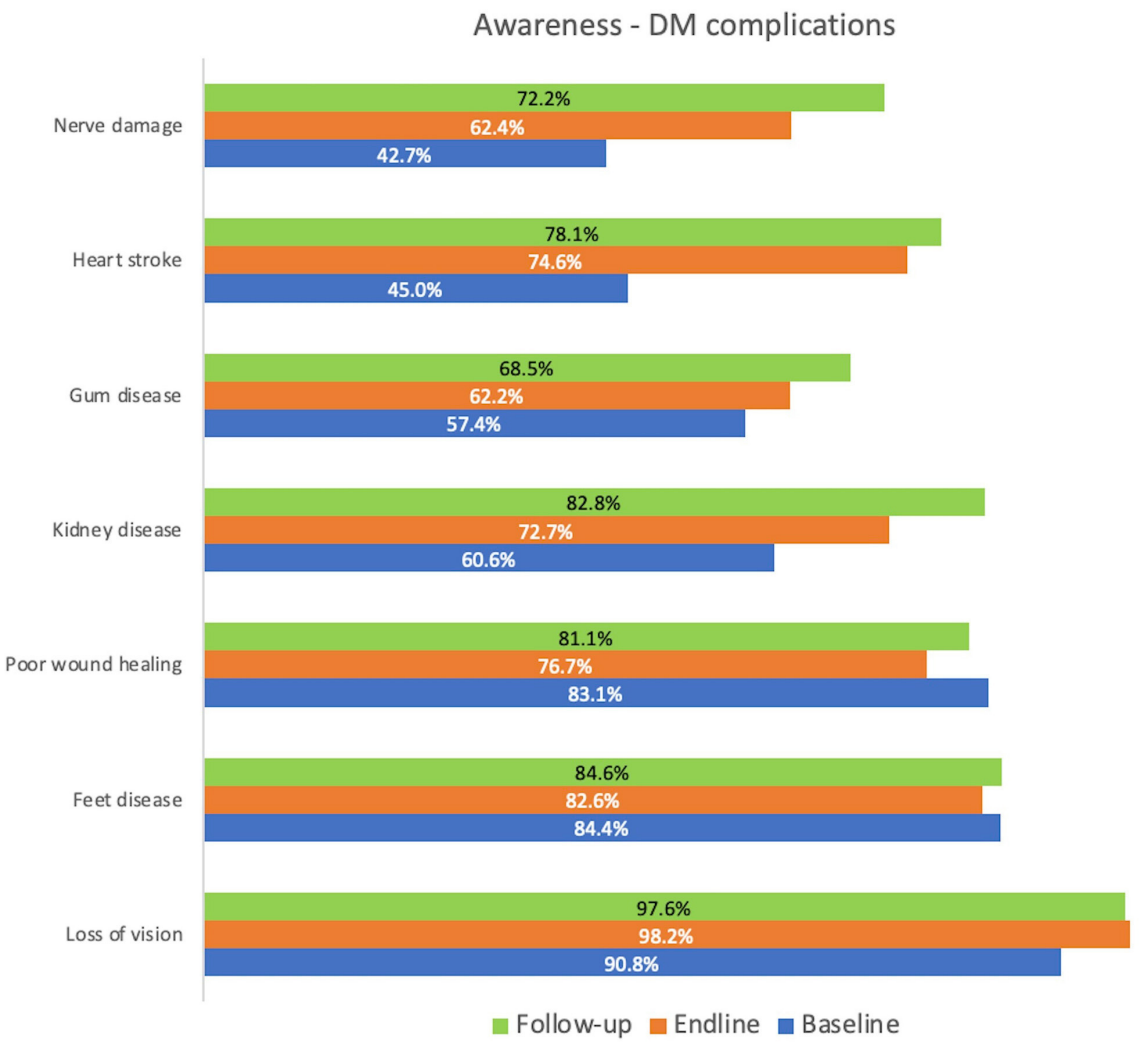
Awareness of diabetes complications.

Figure 4 illustrates the level of awareness among study subjects regarding diabetes complications. The intervention resulted in a substantial increase in awareness of diabetes-related complications, particularly evident in endline and follow-up surveys compared to baseline. Notably, there were significant improvements in awareness regarding complication such as loss of vision (90% to 98%), heart stroke (45% to 78%), and kidney disease (60% to 83%) *p* < 0.001. Additionally, awareness levels of other complications also increased.

**Figure 5:**
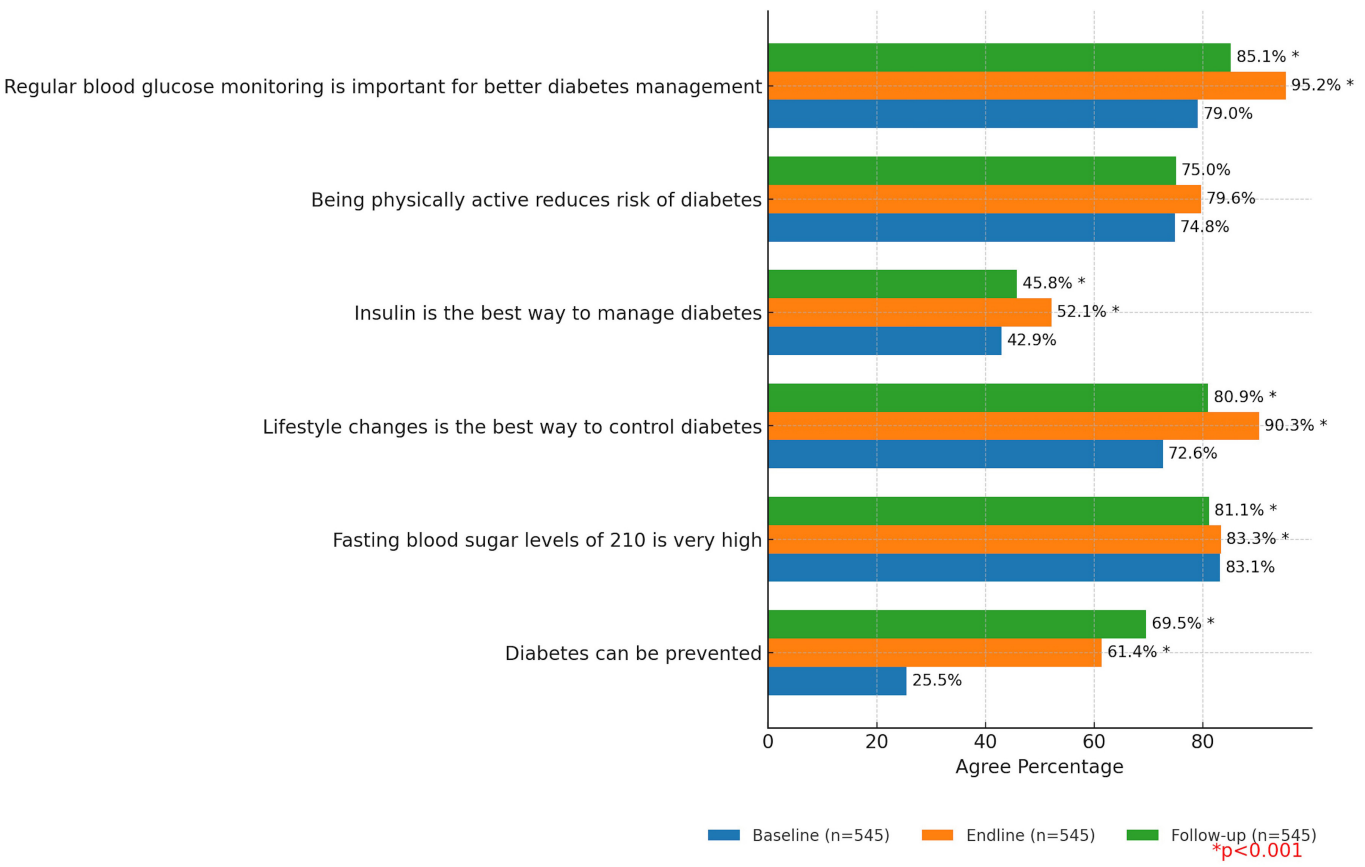
Attitude and perceptions associated with diabetes.

Figure 5 presents the attitudes and perceptions of study participants related to diabetes. The comparative analysis shows significant increases in agreement on key statements across different time points. The belief that diabetes can be prevented saw a remarkable rise from 25.5% (baseline) to 61.4% (endline) and further to 69.5% (follow up). Understanding lifestyle changes as the best way to control diabetes also witnessed a notable increase from from 72.6% (baseline) to 90.3% (endline) and 80.9% (follow up). Moreover, the recognition of regular blood glucose monitoring as essential for better diabetes management showed substantial growth, increasing from 79% (baseline) to 95.2% (endline) and 85.1% (follow up).

### Impact of intervention (diabetes prevention messages)

Over 90% of the participants recalled receiving messages about diabetes prevention in both the endline and follow-up groups (Table 1). Among them, 83% in the endline and 73% in the follow-up group reported making necessary lifestyle changes. The messages had a particularly strong impact on promoting healthy eating habits, encouraging lifestyle changes, and fostering the adoption of medical information adoption, resulting in certain beneficial modifications in the participants' behaviors in both endline and follow-up assessment. In the follow-up group, it was observed that 21.5% of the study participants expressed limitation in their ability to engage in regular walking.

**Table 1:** Impact of diabetes awareness messages on behavioral changes.

**Question**	***n* (%) endline**	***n* (%) follow up**
**Recall of messages (Yes)**		
Healthy eating habits	525 (96%)	515 (94%)
Lifestyle changes	457 (83.5%)	493 (94.5%)
Awareness about diabetes	498 (91.3%)	499 (90.4%)
Fitness/exercising	393 (72.1%)	427 (78.3%)
Medical information about diabetes	520 (95.4%)	526 (96.5%)
**Made changes due to messages (Yes)**	**447 (82%)**	**400 (73.3%)**
**If yes what changes made due to messages**		
Avoiding sweets, eating sugar-free diet, fruits and vegetables taking medicine on time, etc.	213 (39%)	324 (59%)
Started yoga, daily walking, and exercise	126 (23.1%)	103 (18.9%)
Stopped smoking and drinking alcohol	56 (10.2%)	–
Unable to go for walking	–	117 (21.5%)

n = 545. Bold values indicate number of people who made lifestyle changes after receiving voice messages.

### Practice

The practice section involved assessing the dietary habits and physical activity of the study participants.

#### Practice (Dietary habits)

Table 2 shows change in dietary habits of the study participants.

**Table 2:** Change in dietary habits of study participants: a baseline to endline and follow up comparison.

**Question**	**Baseline group (*n* = 545)**	**Endline group (*n* = 545)**	**Follow-up group (*n* = 545)**
**How often do you eat fruits?** ^*^			
Daily	155 (28.4%)	280 (51.4%)	428 (78.5%)
Once a week	179 (32.8%)	263 (48.3%)	61 (11.19%)
2 to 3 times a week	174 (31.9%)	2 (0.4%)	56 (10.3%)
Never	37 (6.7%)	–	–
**Generally, how many fruits/servings of fruits do you eat daily?**			
One	270 (49.5%)	343 (62.3%)	260 (47.7%0
Two	120 (22%)	127 (23.3%)	80 (14.7%)
Three	94 (17.3%)	58 (10.6%)	194 (35.6%)
More than three	5 (0.9%)	9 (1.7%)	6 (1.1%)
None	56 (10.3%)	8 (1.5%)	5 (0.9%)
**How often do you include green vegetables in your diet?** ^*^			
Daily	75 (13.8%)	159 (28.1%)	146 (26.7%)
Once a week	156 (28.6%)	189 (34.7%)	189 (34.7%)
2–3 times a week	286 (52.5%)	188 (34.5%)	205 (37.6%)
Never	28 (5.14%)	9 (1.7%)	5 (0.92%)
**Generally, how many servings of green vegetables do you eat**			
**daily?** ^*^			
One	101 (18.5%)	351 (64.4%)	233 (42.8%)
Two	20 (3.67%)	18 (3.3%)	28 (5.1%)
Three	9 (1.7%)	7 (1.3%)	37 (6.8%)
More than three	6 (1.1%)	–	4 (0.73%)
None	409 (75.05%)	169 (31.0%)	243 (44.6%)
**In the last week, how many times did you eat green vegetables**			
**as per your daily consumption?** ^*^			
Every day	30 (37.8%)	139 (25.5%)	92 (16.88%)
Two days	118 (21.7%)	206 (37.8%)	179 (32.8%)
Three days	185 (33.9%)	81 (14.9%)	259 (47.5%)
More than 3 days	113 (20.7%)	21 (3.8%)	14 (2.6%)
Do not recall	99 (18.2%)	98 (17.9%)	1 (0.1%)
**Do you consistently avoid eating high fat foods like samosas**,			
**vadai, bajji, bondas, etc.?** ^*^			
Yes	201 (36.8%)	381 (69.9%)	369 (67.7%)

^*^p < 0.001.

The endline and follow-up groups showed improvements in healthy eating habits compared to the baseline group. In the endline group, the percentage of participants who reported daily fruit consumption increased from 28.4% at baseline to 51.4%. Additionally, the proportion of participants avoiding high-fat foods rose from 36.8% to 69.9%. In the follow-up group, daily fruit consumption increased from 28.4% at baseline to 78.5%, and the avoidance of high-fat foods increased from 36.8% to 67.7%.

#### Practice (Physical activity)

Table 3 shows comparison of physical activity patterns of study participants among baseline, endline and follow-up groups.

**Table 3:** Physical activity habits and patterns: a comparative study between baseline endline and follow-up group.

**Physical activity**	**Baseline group (*n* = 545)**	**Endline group (*n* = 545)**	**Follow up ( *n* = 545)**
**In the last 1 week how often, you would have done yoga/**			
**running/gym/aerobics** ^*^			
Daily	40 (7.3%)	211 (38.7%)	211 (38.7%)
Alternate days	11 (2.02%)	37 (6.8%)	72 (13.2%)
Selective days/weekends	9 (1.7%)	32 (5.9%)	9 (1.7%)
Never	485 (88.9%)	265 (48.6%)	253 (46.4%)
**In last 1 week how often would you have [Played any outdoor**			
**sports like basketball, football, cricket, swimming, etc.]** ^*^			
Daily	3 (0.5%)	82 (15%)	15 (2.7%)
Alternate days	6 (1.10%)	119 (21.8%)	133 (24.4%)
Selective days/weekends	5 (0.92%)	13 (2.4%)	12 (2.2%)
Never	531 (97.43%)	331 (60.7%)	385 (70.8%)
**In last 1 week how often, you would have walked**			
Daily	382 (70%)	210 (38.5%)	409 (75%)
Alternate days	35 (6.4%)	105 (19.2%)	13 (2.4%)
Selective days/weekends	20 (3.7%)	25 (4.6%)	5 (0.92%)
Never	108 (19.8%)	205 (37.6%)	118 (21.6%)
**On average how long do you exercise?**			
Less than 30 min	49 (8.9%)	259 (47.5%)	81 (14.8%)
30 min	82 (15%)	63 (11.6%)	162 (29.7%)
More than 30 min	184 (33.8%)	104 (19%)	142 (26%)
Cannot say	230 (42.2%)	119 (21.8%)	160 (29.3%)
**Can you tell how often you do the following?**			
**You consciously tend to take stairs instead of using lifts and**			
**escalators** ^*^			
Regularly	140 (25.7%)	274 (50.2%)	326 (59.8%)
Do it sometimes	150 (27.5%)	91 (16.7%)	205 (37.6%)
Do not do at all	255 (46.8%)	180 (33%)	14 (2.6%)
**You prefer to walk down small distances for daily chores**			
Regularly	399 (73.2%)	398 (73%)	462 (84.7%)
Do it sometimes	95 (17.4%)	125 (23%)	77 (14.1%)
Do not do at all	51 (9.4%)	22 (4%)	6 (1.1%)
**You tend to take short walking breaks when working in office/**			
**home** ^*^			
Regularly do it	388 (71.19%)	453 (83.1%)	507 (93%)
Do it sometimes	117 (21.47%)	91 (16.7%)	35 (6.4%)
Do not do at all	40 (7.34%)	1 (0.2%)	3 (0.6%)
Regularly do it	455 (83.49%)	480 (88%)	503 (92.29%)
Do it sometimes	57 (10.46%)	65 (12%)	39 (7.16%)
Do not do at all	33 (6.06%)	–	3 (0.55%)

^*^P < 0.001.

The endline and follow-up groups demonstrated significant improvements in physical activity compared to the baseline group. The data highlights significant changes in behavior across the baseline, endline, and follow-up groups. Daily participation in yoga, running, gym, and aerobics increased notably in the endline (38.7%) and follow-up (38.7%) groups compared to the baseline (7.3%) group (*p* < 0.001). Similarly, there was a significant increase in outdoor sports engagement in the endline (15%) and follow-up (15%) groups compared to the baseline (0.5%) group (*p* < 0.001). Notably, lifestyle behaviors like regular stair usage (59.8%), walking for daily chores (84.7%), and taking short walking breaks (93%) all demonstrated significantly higher percentages in the follow-up group compared to the baseline and endline groups (*p* < 0.001). These findings indicate a clear positive trend in physical activity and lifestyle choices for the Follow-up group, with particular emphasis on their preference for stairs, walking, and short breaks during work activities. Majority 482/545(88%)/subjects in follow-up group attended community awareness meetings conducted by community health workers in study locations.

### Influence of socio-demographic factors on mDiabetes intervention outcomes

Pre- and post-intervention responses did not show any significant gender and age-specific differences, However, education level and occupation influenced some survey outcomes. Participants with education levels of class 10 and above demonstrated greater knowledge of diabetes risk factors (e.g., hypertension and family history) and secondary complications (e.g., foot disease, nerve damage). They also reported notable improvements in healthy behaviors s such as increased consumption of fruits and vegetables, reduced intake of high-fat foods, and greater use of stairs (*P* < 0.05) between baseline and post-intervention surveys.

In terms of occupation, business owners, skilled laborers, and employed participants exhibited higher awareness of secondary complications (e.g., foot disease, heart failure, nerve damage), and more frequent engagement in healthy practices, including increased fruit consumption, avoidance of high-fat foods, and engagement in physical activities like taking short walking breaks and assisting with household tasks compared to unskilled workers and unemployed participants (*P* < 0.05). These trends were consistent across baseline, endline, and follow-up surveys.

## Discussion

The study findings showcased a significant and positive transformation in awareness, attitude, perceptions, and practices related to diabetes prevention among endline and follow-up groups, surpassing the baseline. The combination of mHealth and community health education interventions had a significant impact in increasing awareness about diabetes among the rural population in India.

The intervention's success is evident from a marked improvement in diabetes awareness. In the endline assessment, 97.43% of study participants reported awareness, and in the follow-up survey, the figure increased to 99.63%, compared to the baseline rate of 82.75%. In a prior survey in South India, only 47% of the rural population was found to be aware of diabetes (13). The current study's higher awareness levels at baseline, endline, and follow-up indicate a substantial improvement in study participants awareness.

The intervention had a notable impact on how participants in different groups understood the causes of diabetes. At the baseline (before the intervention), only 51.7% of participants were aware of the link between being overweight or obese and diabetes. After the intervention, this awareness significantly increased to 95% in the endline group and 95.2% in the follow-up group. Similarly, recognition of hypertension as a cause of diabetes rose significantly from 64.9% at baseline to an impressive 88.6% in the endline group, followed by 83.8% in the follow-up group.

Awareness of other factors like poor eating habits, inactive lifestyle, and family history also showed considerable increases in all three groups, highlighting the intervention's effectiveness in enhancing diabetes-related knowledge among participants. However, it's worth noting that awareness of the importance of regular exercise in preventing diabetes saw minimal change across all three groups despite the intervention efforts.

In the ICMR-INDIAB study, knowledge of diabetes risk factors indicated that 59.8% recognized consuming more sweets (eating habits), while overweight or obesity was identified by only 35.5%, family history of diabetes by 17.7%, high blood pressure by 23.2%, lack of physical activity by 16.5%, and mental stress by 12.2% of the general population. Importantly, awareness about risk factors for diabetes was higher in people with known diabetes compared to the general population. In contrast, our study's findings demonstrate a more substantial improvement in diabetes-related knowledge compared to the INDIAB study (14).

The intervention led to a substantial increase in awareness of diabetes-related complications, particularly evident in endline and follow-up surveys compared to baseline. Notably, there were significant improvements in awareness regarding complications such as loss of vision (90% to 98%), heart stroke (45% to 78%), and kidney disease (60% to 83%). Additionally, awareness levels increased for other complications as well. In the INDIAB study, the most commonly reported affected organs were the feet (54.0%), eyes (52.3%), kidneys (36.3%), heart (33.6%), and nerves (18.7%). Other reported complications included lung problems (19.6%), brain diseases (26.6%), and stomach disorders [16.9%; (14)].

The intervention's positive impact extended to participants' attitudes and perceptions associated with diabetes, with substantial increases in agreement on key statements. Participants' belief in diabetes preventability rose from 25.5% to an encouraging 69.5% emphasizing a hopeful mindset. Additionally, understanding the significance of lifestyle changes for diabetes control increased from 72.6% to 80.9%, while the importance of regular blood glucose monitoring saw a notable increase from 79% to 85.1%. In the INDIAB study, it was observed that 56.3% of participants exhibited an awareness of diabetes. Among these participants, 36.8% (*n* = 1,185) indicated “diet” as a preventive measure, while 45.8% (*n* = 1,474) acknowledged that both “diet and exercise” play a role in diabetes prevention (14).

The enhanced levels of awareness regarding diabetes control measures highlighted in our study could potentially be attributed to the implementation of mhealth interventions, as well as community education initiatives. These endeavors took the form of regular awareness meetings and the distribution of informative materials within the project area. These findings highlight the behavior change intervention's effectiveness of the in promoting positive attitudes toward diabetes management and improving knowledge among the study participants, which is in cognizance with several national and international studies (8, 15–18).

For instance, a study in rural Bangladesh demonstrated that mHealth messaging improved T2DM knowledge and awareness, with messages being actively discussed and disseminated. However, sustained behavior change was challenged by social norms and habits, with participants expressing a preference for group discussions over messaging alone. This highlights that while mHealth is valuable as part of multi-component strategies for diabetes prevention, it may be less effective as a stand-alone intervention for addressing complex, socially influenced behaviors (7, 19).

A study in South East India found mobile phone messaging to be effective in reducing the incidence of type 2 diabetes among men with impaired glucose tolerance. Diabetes developed in 18% of the intervention group compared to 27% in controls (HR 0.64, 95% CI 0.45–0.92; *p* = 0.015), demonstrating that messaging can be a practical and acceptable method for supporting lifestyle modifications in high-risk individuals (20). These examples further validate the positive impact of mHealth interventions in enhancing diabetes prevention and management efforts.

The study's results unveiled the significant influence of diabetes awareness messages on behavioral changes. Over 90% of the participants recalled receiving messages about diabetes prevention in both the endline and follow-up groups. Among them, 83% in the endline and 73% in the follow-up group reported making necessary lifestyle changes. The participants' healthy eating habits, lifestyle choices, and medical information adoption were positively influenced, leading to beneficial behavioral modifications. While the majority showed impressive progress, it is important to acknowledge that the follow-up group encountered certain challenges, with 21.5% of participants reporting limitations in their ability to go for a walk, suggesting potential obstacles in implementing certain behavioral changes in this subgroup. This finding underscores the importance of addressing individual barriers and customizing interventions to accommodate diverse needs (21–23).

The study participants in both the endline and follow-up groups demonstrated remarkable improvements in healthy eating habits compared to the baseline group. Daily fruit consumption increased significantly, with the endline group showing an increase from 28.4% to 51.4% and the follow-up group reaching an inspiring 78.5%. Additionally, consistent avoidance of high-fat foods also saw improvements, with the endline group rising from 36.8% to 69.9% in the follow-up group reaching an 67.7%. These findings reveal the intervention's positive impact of the intervention in fostering healthier dietary choices among the study participants. Lin et al. demonstrated a notable decrease in HbA1c levels and an improvement in healthy diet after the 6 month health coaching. Patients in the intervention group reduced their daily intake of whole grains, fruits, meats, and proteins, as well as fats and oils, while increasing their intake of vegetables (24). Similar health coaching sessions involving goal setting, community education, and telephone and mobile app interventions have demonstrated a positive impact in various studies (25–29). Lifestyle medicine is emerging as a pivotal discipline in the management of chronic diseases like diabetes. The practice of lifestyle medicine necessitates proficiency in addressing multiple health risk behaviors and enhancing self-management. This entails targeting areas such as diet, physical activity, behaviors change, body weight control, adherence to treatment plans, stress and coping, spirituality, mind-body techniques, and tobacco and substance abuse (30). Our study findings shed light on some of these key areas.

Physical activity levels showed a notable increase in both the endline and follow-up groups when compared to the baseline group. Participation in a variety of physical activities, including exercise, yoga, running, gym, aerobics, and outdoor sports, increased significantly, with *p*-values indicating highly significant changes (*p* < 0.0001). The follow-up group demonstrated additional positive behaviors, such as choosing stairs over elevators, walking for daily tasks, and incorporating walking breaks during work, indicating sustained and improved adoption of physical activity. These findings underscore the effectiveness of the intervention in promoting an active and healthier lifestyle among the study participants. The positive impact of physical activity interventions on diabetes prevention and management has been highlighted in diverse studies (31–33).

Our results indicated that age and gender did not significantly impact the effectiveness of the mDiabetes intervention. However, intervention was more impactful among participants with higher levels of education and employment status, particularly in improving knowledge of diabetes-related complications, and lifestyle determinants such as dietary habits and physical activities as observed in both baseline and post-intervention surveys. Supporting evidence from a cross-sectional analysis of 44 LMICs underscores the critical role of education in diabetes prevention. Individuals with secondary schooling were 6.5% points more likely to receive dietary counseling and 21.3% points more likely to undergo blood glucose screening compared to those with no formal education (34). Similarly, data from India's National Family Health Survey (NFHS-5, 2019–2021) reveal stark socioeconomic disparities, with individuals in the highest wealth quintile being significantly more likely to be aware of, treated for, and have their diabetes under control compared to those in the lowest quintile (*p* < 0.001). Diabetes awareness, treatment, and control (ATC) remain notably low among poorer and less educated groups (35).

The mDiabetes program by Arogya World demonstrated a 15% cumulative improvement in diabetes risk behaviors among 1 million participants over 6 months through 56 text messages, promoting increased exercise, and fruit, and vegetable intake (8). Similarly, the myArogya app implemented at Arogya World Healthy Workplaces in Bengaluru, led to significant reductions in HbA1c and blood pressure levels and increased physical activity among prediabetic individuals (9). A television-based lifestyle intervention for high-risk T2D patients across three cities improved cardiometabolic risk factors, physical activity, dietary habits, and weight loss, with higher engagement in video content, yielding better outcomes (10). Furthermore, a randomized controlled trial on personalized text messaging for newly diagnosed T2D patients demonstrated reductions in HbA1c and LDL-c, highlighting the importance of sustained behavioral change (36). These findings align with our study's results, demonstrating the effectiveness of mHealth interventions in improving diabetes awareness, addressing risk factors, and promoting behavioral changes, underscoring mHealth as a scalable tool for diabetes prevention and management.

## Strengths and limitations of the study

The strengths of the present study encompass a comprehensive approach to creating awareness about diabetes prevention and its complications through a multipronged strategy. This encompassed utilizing m-health interventions, community education in the form of awareness meetings, and distribution of informational materials, effectively reaching a population of over 100,000 in the project area. By conducting interviews with the same participants at baseline, endline, and follow-up, the longitudinal data provided valuable insights into knowledge, attitudes, and practices related to diabetes prevention. However, the study also has its limitations. Generalizing the findings to regions with diverse socio demographics, such as urban areas or populations with varied cultural and ethnic backgrounds, may be challenging. Additionally, the absence of objective measurements like blood sugar levels, body weight, and body mass index limits the ability to fully assess the impact of the interventions. Digital literacy of participants was not assessed, which could have influenced the effectiveness of the mHealth interventions. Furthermore, the data collected is based on self-assessments and participant evaluations, lacking validation from health institutions or clinical records to confirm actual reductions in health issues. Finally, while “community awareness” is inferred from aggregated individual responses, direct measures of community-level awareness, such as qualitative assessments or public institution interviews, were not conducted.

## Conclusion

The integrated mHealth and community health education intervention proved to be a game changer in diabetes prevention among the rural population in India. The significant improvements in awareness, dietary habits, and physical activity among the study participants showcase the intervention's effectiveness in fostering preventive behaviors and encouraging healthy lifestyle changes. The sustained and enhanced physical activity adoption observed in the follow-up group further highlights the potential long-term impact of the intervention. These study serves as a valuable blueprint for future public health initiatives aimed at tackling diabetes prevention resource-limited settings, offering hope and better health prospects for the communities involved.

## Data Availability

The original contributions presented in the study are included in the article/Supplementary material, further inquiries can be directed to the corresponding author/s.
