# Supplementary material for: Effectiveness of mDiabetes intervention in enhancing diabetes awareness and promoting healthy lifestyle changes among the general population in rural India
Source: Front Public Health. 2025 Jan 29;12:1470615. doi: 10.3389/fpubh.2024.1470615 (PMC11818752; doi:10.3389/fpubh.2024.1470615)
Supplement: Supplementary file 1 [file Data_Sheet_1.docx]

#### **Content theme and examples of m diabetes voice messages**

1. **Medical Information (20 messages)**
   - Focus: Complications of diabetes, risk factors, importance of testing, and medical advice.
   - Examples:
     - *"High blood sugar from diabetes can cause problems in your eyes, kidneys, heart, feet, and nerves. These are called diabetes complications."*
     - *"If you have diabetes, visit an eye doctor regularly to prevent blindness caused by diabetic retinopathy."*
2. **Lifestyle Changes (8 messages)**
   - Focus: Importance of physical activity, quitting smoking, and adopting healthy habits.
   - Examples:
     - *"The good news is that type 2 diabetes can be prevented by physical activity and eating healthy foods."*
     - *"If you smoke, especially with diabetes, take steps to quit. Ask family and friends for support."*
3. **Nutrition (13 messages)**
   - Focus: Eating fresh vegetables, fruits, and whole grains while avoiding sugary, fried, and oily foods.
   - Examples:
     - *"Healthy eating means eating plenty of fresh vegetables, fruits, and avoiding sugary or oily foods like namkeens and sweets."*
     - *"Instead of fried snacks, eat a handful of nuts or fruits as a healthy alternative."*
4. **Fitness and Physical Activity (9 messages)**
   - Focus: Daily exercise such as walking, yoga, and climbing stairs.
   - Examples:
     - *"Walk briskly or exercise for 30 minutes daily to manage blood sugar and avoid diabetes."*
     - *"Start with 10 minutes of walking 2 days a week, and gradually increase to 30 minutes daily."*
5. **Motivational and Awareness Messages (6 messages)**
   - Focus: Encouraging participants to take control of their health and share awareness.
   - Examples:
     - *"By receiving these messages, you are learning to improve your health and live better with diabetes."*
     - *"To live a healthy life, learn as much as you can about diabetes and how to manage it through healthy eating and physical activity."*
