# Supplementary material for: Effectiveness of mDiabetes intervention in enhancing diabetes awareness and promoting healthy lifestyle changes among the general population in rural India
Source: Front Public Health. 2025 Jan 29;12:1470615. doi: 10.3389/fpubh.2024.1470615 (PMC11818752; doi:10.3389/fpubh.2024.1470615)
Supplement: Supplementary file 2 [file Data_Sheet_2.docx]

| **Variables** | **Participants (n=545)** | **Non-participants (n=474)** | **P-value** |
| --- | --- | --- | --- |
| **Demographics** |  |  |  |
| Age (Years) | 19-85 | 24-95 | 0.105 |
| Gender Male/Female | 234 (42.9%) / 311 (57.1%) | 214 (45.1%) / 260 (54.9%) | 0.478 |
| Education  (No schooling)  (Primary-High school)  (Degree) | 242(44.4%)  283(51.9%)  20(3.7%) | 188(39.7%)  244(51.5%)  42(8.87%) | 0.118 |
| **Diabetes Awareness** |  |  |  |
| Yes/No | 450 (82.6%) / 95 (17.4%) | 387 (81.6%) / 87 (18.4%) | 0.701 |
| **Awareness of Risk Factors and Complications** |  |  |  |
| Obesity Yes/No | 283 (51.9%) / 262 (48.1%) | 235 (49.6%) / 239 (50.4%) | 0.454 |
| Hypertension Yes/No | 352 (64.6%) / 193 (35.4%) | 336 (70.9%) / 138 (29.1%) | 0.032 |
| Poor eating habits Yes/No | 420 (77.1%) / 125 (22.9%) | 324 (68.4%) / 150 (31.6%) | 0.002 |
| Heart Failure Yes/No | 244(44.8 ) / 301 (55.2%) | 206 (43.5%) / 268 (56.5%) | 0.674 |
| Vision Loss Yes/No | 494 (90.6%) / 51 (9.4%) | 405 (85.4%) / 69 (14.6%) | 0.010 |
| Nerve Damage Yes/No | 233 (42.8%) / 312 (57.2%) | 168 (35.4%) / 306 (64.6%) | 0.017 |
| **Awareness of Lifestyle Habits to Prevent Diabetes** |  |  |  |
| Fruits intake in diet Low/Med/High | 155 / 353 / 37 (28.4% / 64.7% / 6.8%) | 152 / 273 / 49 (32.1% / 57.6% / 10.3%) | 0.039 |
| Vegetables intake in diet Low/Med/High | 101 / 32 / 409 (18.5% / 6.5% / 75.0%) | 100 / 43 / 331 (21.1% / 9.1% / 69.8%) | 0.284 |
| High-Fat contained food habits Yes/No | 200 (36.7%) / 345 (63.3%) | 204 (43.0%) / 270 (57.0%) | 0.039 |
| Exercise Yes/No | 103 (18.9%) / 442 (81.1%) | 86 (18.1%) / 388 (81.9%) | 0.757 |
| Walking upstairs Yes/No | 141(25.9% ) / 148 (27.2%) | 119(25.1% ) / 129 / (27.2%) | 0.958 |

**Supplementary table: Comparative Analysis of Baseline Responses Between Participants (n=545) and Non-Participants (n=474)**
