# Supplementary figures and images for: Effectiveness of mDiabetes intervention in enhancing diabetes awareness and promoting healthy lifestyle changes among the general population in rural India
Source: Front Public Health. 2025 Jan 29;12:1470615. doi: 10.3389/fpubh.2024.1470615 (PMC11818752; doi:10.3389/fpubh.2024.1470615)

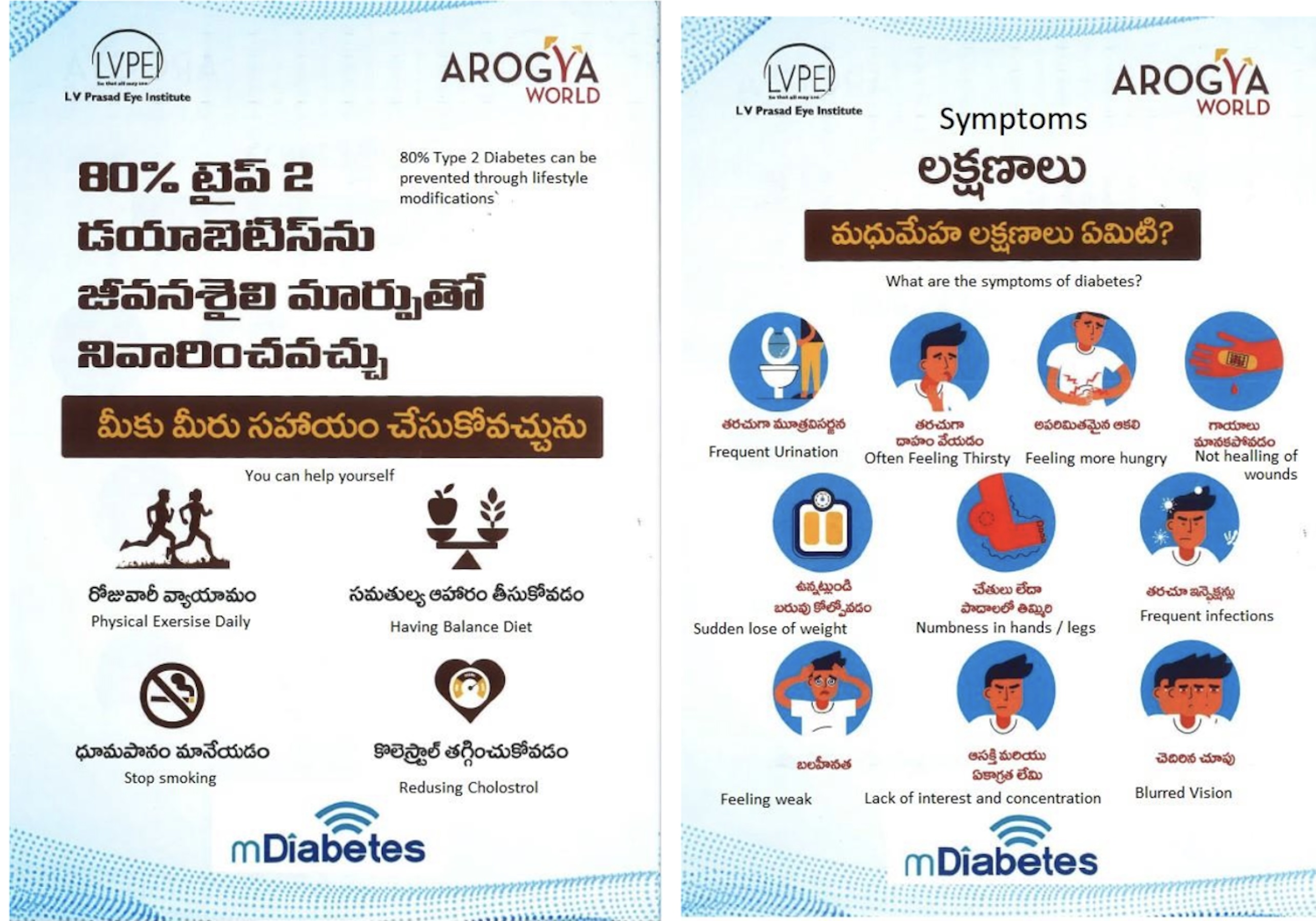

Supplement: Supplementary file 3 [file Image_1.jpeg]
